# Optimization of a Bovine Intracytoplasmic Sperm Injection System Using Sex-Sorted Sperm: Screening with Slaughterhouse-Derived Oocytes and Validation with Ovum Pick-Up-Derived Oocytes

**DOI:** 10.3390/ani16192983

**Published:** 2026-09-22

**Authors:** Xuexiao Song, Tao Zhang, Hongxing Yang, Heqiang Li, Baobao Zhao, Yanfang Wu, Zheng Wang, Dexiang Fan, Yaochang Wei, Dong Zhou, Yongsheng Wang

**Affiliations:** 1 Department of Clinical Veterinary Medicine, College of Veterinary Medicine, Northwest A&F University, Xianyang 712100, China; 2022065036@nwafu.edu.cn (X.S.); zt2025060374@nwafu.edu.cn (T.Z.); 2025055645@nwafu.edu.cn (H.Y.); lihq@nwafu.edu.cn (H.L.); 2020050571@nwsuaf.edu.cn (B.Z.); wyf18829353119@163.com (Y.W.); anhuiwangzheng@nwafu.edu.cn (Z.W.); 2022055585@nwafu.edu.cn (D.F.); 18428374393@163.com (Y.W.); 2 Key Laboratory of Animal Biotechnology of the Ministry of Agriculture and Rural Affairs, Northwest A&F University, Xianyang 712100, China

**Keywords:** sex-sorted sperm, OPU, ICSI, dairy cow, ethanol activation, pronuclear formation

## Abstract

Sex-sorted sperm permit the preferential production of offspring of a specified sex, but flow sorting and cryopreservation can compromise sperm function and reduce the efficiency of conventional in vitro fertilization (IVF). We sequentially evaluated oocyte maturation duration, sperm processing, post-injection incubation medium, and nine activation regimens using slaughterhouse-derived oocytes, and then evaluated the best-performing combination under the tested sequential conditions using ovum pick-up (OPU)-derived oocytes. In conventional IVF, the presumptive normal fertilization rate, based on the presence of two pronuclei (2PN), was 2.45% with X-sorted sperm and 46.13% with conventional sperm. The final selected combination, consisting of 22 h maturation, Brackett–Oliphant (BO)-based swim-up, oocyte maturation medium (OM) during post-injection incubation, and treatment with 7% ethanol for 5 min [ETH (5)], resulted in a presumptive normal fertilization (2PN) rate of 52.92%, a blastocyst rate of 10.46%, and an ICM/TE ratio of 0.82. In the validation using OPU-derived oocytes, the blastocyst formation rate after ssICSI was 13.46% when calculated relative to injected oocytes and 16.84% when calculated relative to surviving oocytes. These rates differed significantly from the blastocyst formation rate after ssIVF (7.88%), whereas cleavage rates did not differ significantly between groups. ETH (5) provided the most favorable in vitro balance of presumptive 2PN formation, blastocyst development, and ICM/TE cell allocation among the tested regimens. Because the factors were optimized sequentially, their interactions were not estimated, which represents a limitation of this study. Furthermore, implantation, pregnancy, and calving rates require validation in independent in vivo embryo-transfer experiments.

## Simple Summary

Flow-cytometric sex sorting enables the preferential production of female calves but can impair sperm function and reduce the efficiency of conventional in vitro fertilization (IVF). We therefore evaluated four components of bovine intracytoplasmic sperm injection (ICSI): oocyte maturation time, sperm processing, the post-injection incubation medium, and the activation protocol. The factors were screened sequentially with slaughterhouse-derived oocytes, and the best-performing combination under the tested sequential conditions was subsequently evaluated with ovum pick-up (OPU)-derived oocytes. In the final OPU validation, sex-sorted-sperm ICSI (ssICSI) improved second-polar-body extrusion and blastocyst formation relative to sex-sorted-sperm IVF (ssIVF), whereas cleavage was comparable between the groups. Compared with the other activation regimens, exposure to 7% ethanol for 5 min [ETH (5)] resulted in a favorable inner cell mass-to-trophectoderm (ICM/TE) cell allocation. These findings are limited to in vitro outcomes; embryo transfer, pregnancy, and calving competence remain to be evaluated.

## 1. Introduction

Intracytoplasmic sperm injection (ICSI) introduces a single spermatozoon directly into the oocyte cytoplasm [1,2]. Because it requires very few spermatozoa [3], ICSI is particularly valuable when semen from genetically important bulls is scarce or costly. Flow cytometry separates X- and Y-chromosome-bearing spermatozoa and thereby permits sex-preselected offspring [4,5,6,7], an economically important application in dairy production. However, staining, high-pressure sorting, and cryopreservation can impair sperm motility and fertilizing capacity [8,9,10], thereby limiting the efficiency of conventional IVF with sex-sorted sperm [11]. One study found that blastocyst production after sex-sorted-sperm ICSI approached that obtained with conventional-sperm ICSI [12], consistent with related reports [13,14]. On the maternal side, OPU enables repeated recovery of oocytes from living, genetically valuable donors [15,16], although OPU-derived oocyte competence can vary [17]; lower-quality oocytes consequently show reduced blastocyst development [18]. Conventional IVF with sex-sorted sperm remains inefficient [19,20], whereas ICSI can improve the use of each available gamete when both sperm and oocytes are limited [21,22].

Despite continued refinement of bovine ICSI [23,24,25,26], protocols for the combined use of X-sorted sperm and OPU-derived oocytes remain incompletely characterized. Oocyte maturation duration can influence cytoplasmic competence and subsequent development [27,28], but early pronuclear events have seldom been incorporated into maturation-time comparisons [29,30]. Sperm preparation may also require specific consideration [31,32]. Heparin–glutathione pretreatment improved conventional-sperm ICSI in one study but reduced cleavage and blastocyst formation with sex-sorted sperm [33], indicating that sorting history can modify the response to sperm processing [34,35]. Post-injection incubation medium has generally been treated as a fixed procedural detail rather than an experimental factor. Bovine oocytes often respond incompletely to injection alone [36] and therefore require exogenous activation for acceptable cleavage [37]; ethanol activation has also been associated with different post-transfer outcomes from ionomycin plus 6-DMAP in a PIEZO-assisted system [38]. Activation regimens for sex-sorted-sperm ICSI remain incompletely compared at the pronuclear and blastocyst-lineage levels [39,40]. Blastocyst cell allocation, including ICM and TE cell numbers, provides a useful in vitro quality descriptor [41], but it is a surrogate endpoint and cannot establish implantation or calving competence.

We therefore used pronuclear configuration together with embryo-development outcomes to screen sequential changes in maturation duration, sperm processing, post-injection incubation medium, and activation treatment. Pronuclear categories included presumptive normal fertilization (2PN), a partially decondensed sperm head at the observation time, condensed-sperm-head configurations, abnormal pronuclear numbers, and MII-arrested/unactivated oocytes. Day-7 blastocyst cell allocation was then evaluated by immunofluorescence. The objective was to identify a reproducible, best-performing combination under the tested sequential conditions.

Experiment 1 used a 2 × 2 factorial IVF design to evaluate oocyte source (slaughter-house-derived or OPU-derived) and sperm type (conventional or X-sorted). Experiment 2 sequentially screened maturation time (18–26 h), sperm preparation (BO swim-up or Percoll), incubation medium (OM or IVF medium), and nine activation regimens using slaughter-house-derived oocytes. Experiment 3 compared blastocyst cell allocation across selected activation groups. Experiment 4 used OPU-derived oocytes to compare the complete, sequentially selected ssICSI protocol with ssIVF.

## 2. Materials and Methods

Unless otherwise stated, all chemicals were purchased from Sigma-Aldrich (St. Louis, MO, USA).

### 2.1. Farms and Animals

The study was conducted on large commercial dairy farms in Ningxia, China. Forty clinically healthy, multiparous Holstein cows were used as OPU donors. Donors were 3–6 years old, in parities 2–4, lactating, and more than 40 days in milk at the start of the study. Each donor underwent three OPU sessions separated by at least 15 days. All donor cows underwent the same ovarian stimulation protocol that included dominant-follicle aspiration, prostaglandin F2α (25 mg, intramuscularly; Ningbo Sansheng, Ningbo, China), and follicle-stimulating hormone (100 IU, intramuscularly every 12 h for five administrations; Ningbo Sansheng). OPU was performed 36 h after the final FSH administration. Animals were maintained in free-stall housing, fed a total mixed ration three times daily, and had unrestricted access to water. All procedures were approved by the Animal Welfare and Ethics Committee of Northwest A&F University (Approval No. 2021028) and complied with national animal-welfare requirements.

### 2.2. Collection and In Vitro Maturation of Oocytes

OPU-derived oocytes (OPU-O) were recovered under transvaginal B-mode ultrasonographic guidance. Follicular fluid was collected into a collection cup, filtered, and repeatedly rinsed with saline until the filtrate was clear. Cumulus-oocyte complexes (COCs) were identified under a stereomicroscope and washed in HEPES-buffered medium containing 6.669 mg/mL NaCl, 0.239 mg/mL KCl, 0.168 mg/mL NaHCO_3_, 0.048 mg/mL NaH_2_PO_4_·H_2_O, 1 µL/mL sodium lactate, 0.294 mg/mL CaCl_2_·2H_2_O, 0.102 mg/mL MgCl_2_·6H_2_O, 2.384 mg/mL HEPES, 3.0 mg/mL BSA, and 0.022 mg/mL sodium pyruvate. COCs with an intact oolemma, homogeneous cytoplasm, and compact multilayered cumulus were retained; COCs showing degeneration, membrane disruption, cytoplasmic fragmentation, or an overmature/markedly expanded appearance were excluded. Eligible COCs from each donor/session were randomly and as evenly as possible allocated among contemporaneous treatment groups. The OM was based on TCM-199 and contained 10% fetal bovine serum, 1% insulin-transferrin-selenium, 0.022 mg/mL sodium pyruvate, 14.16 µL/mL sodium lactate, and 0.75 IU/mL human menopausal gonadotropin (HMG). Maturation culture was performed at 38.5 °C under 5% CO_2_, 7% O_2_, and saturated humidity.

For slaughterhouse-derived oocytes (SL-O), ovaries were transported to the laboratory at a controlled temperature. Excess connective tissue was removed; ovaries were cleaned with 75% ethanol and rinsed repeatedly with saline. Follicular contents were aspirated with a syringe, and COCs were recovered under a stereomicroscope.

Recovered COCs were washed in the HEPES-buffered medium described above and graded using the same morphological inclusion and exclusion criteria as OPU-derived COCs. Eligible COCs were rinsed once in OM and matured in OM at 38.5 °C under 5% CO_2_, 7% O_2_, and saturated humidity. Unless otherwise specified, the medium volume was adjusted to 10 µL per oocyte.

### 2.3. Sperm Processing

Commercial X-sorted frozen semen from three Holstein bulls and commercial conventional frozen semen from three different Holstein bulls were used. Each semen type comprised three coded commercial batches, with one batch corresponding to each bull. The X-sorted semen contained X-chromosome-bearing spermatozoa; the supplier reported a commercial sorting purity of at least 90%, although batch-specific purity was not independently verified in this study. Within each independent run, comparisons involving a given semen type used the same bull and coded batch across contemporaneous treatment groups. For each IVF procedure using conventional semen, one 0.25 mL conventional-semen straw was thawed. For each ICSI procedure, one 0.25 mL X-sorted-semen straw was thawed, whereas two X-sorted-semen straws were thawed for each ssIVF procedure. Final sperm concentrations were adjusted to 1 × 10^4^ spermatozoa/mL for ICSI and 2 × 10^6^ spermatozoa/mL for IVF. Because only three bulls and three coded batches were available for each semen type, bull and batch effects could not be estimated reliably as random effects; possible confounding between semen type and the distinct bull sets is therefore acknowledged.

For BO swim-up, 4 mL BO medium (SOF supplemented with 0.005 mg/mL sodium heparin) and 2 mL HEPES wash medium were equilibrated for at least 1 h. After thawing at 38 °C, semen was gently placed at the bottom of a tube containing 4 mL BO. The tube was held at 45° for 30 min at 38.5 °C under 5% CO_2_, 7% O_2_, and saturated humidity. The upper fraction was collected and centrifuged at 500× *g* for 10 min at room temperature. The pellet was resuspended in 1 mL HEPES wash medium and washed twice at 300× *g* for 5 min at room temperature.

For Percoll density-gradient separation, 1 mL PureSperm 80 (PS40-100, Nidacon International AB, Gothenburg, Sweden) was overlaid with 1 mL PureSperm 40 (PS40-100, Nidacon International AB, Gothenburg, Sweden) in a 15-mL tube and equilibrated for at least 1 h together with 4 mL HEPES wash medium. Thawed semen was layered above the gradient and centrifuged at 500× *g* for 10 min at room temperature. The pellet was recovered, resuspended in 4 mL HEPES wash medium, and washed once at 300× *g* for 5 min at room temperature.

### 2.4. In Vitro Fertilization (IVF)

Oocytes matured for 24 h were washed in IVF medium and transferred to IVF-medium droplets (IVF Bioscience, Falmouth, UK) at 10 µL per oocyte. Prepared spermatozoa were added to a final concentration of 2 × 10^6^ /mL spermatozoa. Fertilization proceeded for 12–18 h at 38.5 °C under 5% CO_2_, 7% O_2_, and saturated humidity. Cumulus cells were then removed by gentle vortexing or pipetting in SOF containing 6.545 mg/mL NaCl, 0.2997 mg/mL KCl, 0.3308 mg/mL CaCl_2_·2H_2_O, 0.1057 mg/mL MgCl_2_·6H_2_O, 3.108 mg/mL NaHCO_3_, 2.504 mg/mL glucose, 0.1376 mg/mL sodium pyruvate, and 3 mg/mL BSA. Presumptive zygotes were cultured in IVC medium (IVF Bioscience) at 38.5 °C under 5% CO_2_, 7% O_2_, and saturated humidity. Cleavage was assessed 48 h after insemination, and blastocyst formation was assessed on day 7.

### 2.5. Intracytoplasmic Sperm Injection (ICSI)

ICSI manipulation and sperm-handling microdroplets were prepared in a 35 mm dish using Gamete Buffer (K-SIGB-20, Cook Medical, Brisbane, QLD, Australia) and polyvinylpyrrolidone (PVP; Vitrolife, Gothenburg, Sweden), respectively. Procedures were performed on an inverted microscope (IX73; Olympus, Tokyo, Japan) fitted with Narishige micromanipulators (Narishige Co., Ltd., Tokyo, Japan). The holding pipette had an inner diameter below 20 µm and an outer diameter of 150–200 µm; the injection pipette had an inner diameter of 6–8 µm. Holding and injection pipettes were prepared from BJ-40 glass (Beijing, China) and borosilicate capillary tubing (B100-75-15; Sutter Instrument, Novato, CA, USA), respectively.

A morphologically intact spermatozoon without adherent debris was selected in PVP, and its tail was mechanically severed before aspiration into the injection pipette. The oocyte was held with the first polar body at the 12-o’clock position and injected from the 3-o’clock position. The pipette was advanced approximately two-thirds across the oocyte, and a small amount of cytoplasm was aspirated until membrane breakage was evident; the spermatozoon and aspirated cytoplasm were then returned to the ooplasm. All ICSI procedures were performed by the same experienced operator. The number of oocytes surviving injection was defined as the number with an intact plasma membrane and no cytoplasmic leakage at the end of the recovery period. After recovery, oocytes with an incomplete plasma membrane, cytoplasmic leakage, or obvious degeneration were excluded, and the number of surviving oocytes was recorded.

### 2.6. Activation Treatment

Immediately after ICSI, oocytes were recovered in OM for 20 min at 38.5 °C under 5% CO_2_, 7% O_2_, and saturated humidity. The protocol-specific treatments described below were then initiated. The incubation medium used during activation was OM or IVF medium. OM used during recovery and incubation had the composition specified in Section 2.2.

(1) ETH (5): oocytes remained in incubation medium until 3–4 h after ICSI and were then exposed to 7% ethanol in OM for 5 min.
(2) ETH (10): oocytes remained in incubation medium until 3–4 h after ICSI and were then exposed to 7% ethanol in OM for 10 min.
(3) ION + ETH: oocytes were exposed to 5 µM ionomycin in SOF for 5 min, washed thoroughly in SOF, incubated in incubation medium until 3–4 h after ICSI, and then exposed to 7% ethanol in OM for 5 min.
(4) ION*2 + ETH: oocytes were exposed to 5 µM ionomycin in SOF for 5 min, incubated for 30 min, exposed a second time to 5 µM ionomycin for 5 min, washed thoroughly in SOF, incubated in incubation medium until 3–4 h after ICSI, and then exposed to 7% ethanol in OM for 5 min.
(5) ION + 6D: oocytes were exposed to 5 µM ionomycin in SOF for 5 min, washed thoroughly in SOF, incubated in incubation medium for 3–4 h, and then exposed to 2 µM 6-dimethylaminopurine (6-DMAP) in SOF for 4 h.
(6) ION*2 + 6D: oocytes were exposed to 5 µM ionomycin in SOF for 5 min, incubated in incubation medium for 30 min, exposed a second time to 5 µM ionomycin for 5 min, washed thoroughly in SOF, and then exposed to 2 µM 6-DMAP in SOF for 4 h.
(7) ION: oocytes were exposed to 5 µM ionomycin in SOF for 5 min, washed thoroughly in SOF, and incubated for 5 h.
(8) ION*2: oocytes were exposed to 5 µM ionomycin in SOF for 5 min, washed in SOF, incubated in incubation medium for 5 h, exposed a second time to 5 µM ionomycin for 5 min, washed again, and incubated for a further 5 h.
(9) Electrical activation (EA): after a 1 min wash in activation solution, oocytes received one direct-current pulse (1.6 kV/cm for 20 µs) in a pre-equilibrated activation chamber and were then washed twice in SOF.

Except for the specific comparison between OM and IVF medium, post-injection recovery and protocol-specific incubation were conducted in OM. All recovery and activation incubations were performed at 38.5 °C under 5% CO_2_, 7% O_2_, and saturated humidity.

### 2.7. In Vitro Culture (IVC)

After activation, oocytes were washed in SOF and cultured at 38.5 °C under 5% CO_2_, 7% O_2_, and saturated humidity. Second polar body (2PB) extrusion was assessed 8 h after the start of culture and was interpreted as evidence of meiotic completion/oocyte activation. Cleavage and blastocyst formation were assessed at 48 h and 168 h, respectively. For ICSI groups, survival was calculated relative to the number of initially injected oocytes. Subsequent developmental outcomes in the sequential-screening analyses were calculated relative to surviving post-injection oocytes to focus on developmental competence after technically successful injection and to reduce variation attributable to injection-pipette characteristics or manipulation-related loss. In the endpoint OPU validation, outcomes were additionally calculated relative to initially injected oocytes so that overall production efficiency could be compared with the number of oocytes initially allocated to ssIVF. Both denominator definitions are stated explicitly in the corresponding captions.

### 2.8. Assessment of Pronuclear Formation

Because pronuclear morphology changes dynamically, a preliminary timing assessment examined oocytes at 8, 10, 12, 14, 16, and 18 h after ICSI (Table S1). At 16 h, the frequency of condensed sperm heads had reached a low plateau, most oocytes with presumptive normal fertilization displayed distinct pronuclei, MII-arrested oocytes remained distinguishable, and extensive cleavage had not yet occurred; by 18 h, cleavage increasingly interfered with classification. Therefore, 16 h was selected as the standardized assessment time because it provided a relatively clear practical distinction between presumptive normal fertilization and abnormal or presumptively delayed configurations. At 16 h, the zona pellucida was removed with 0.2% chymotrypsin, and oocytes were fixed in 4% paraformaldehyde (Beyotime, Shanghai, China) for 4 h, washed in PBS-PVA, stained with DAPI (C1005; Beyotime, Shanghai, China) for 10 min in the dark, washed again, and examined by fluorescence microscopy. The following mutually exclusive morphological categories were recorded: presumptive normal fertilization, defined as one male and one female pronucleus (2PN); presumptive delayed fertilization, defined morphologically as one pronucleus with a partially decondensed sperm head at 16 h (1PN + PDSH); condensed-sperm-head configurations (1PN + CSH or 2PN + CSH); abnormal pronuclear number (1PN alone or ≥3PN); and MII-arrested/unactivated morphology, with or without a condensed sperm head. Percentages were calculated per stained oocyte. Because classification was based on morphology at one fixed time point and paternal origin was not verified, rare temporal misclassification cannot be excluded.

### 2.9. Immunofluorescence Staining

Day-7 expanded blastocysts of Gardner grades AA, AB, or BA were randomly coded before staining. Blastocysts were sampled from independent experimental runs for every treatment. Blastocysts were fixed in 4% paraformaldehyde, permeabilized with 0.5% Triton X-100 (Beyotime, Shanghai, China) for 40 min, blocked for 4 h at room temperature, and incubated overnight at 4 °C with rabbit anti-SOX2 (1:200; CST-D6D9) and mouse anti-CDX2 (1:200; AM392). After washing, samples were incubated with Alexa Fluor 488- and Alexa Fluor 555-conjugated secondary antibodies (1:800; Beyotime, Shanghai, China) for 2 h in the dark and counterstained with DAPI for 10 min. Total nuclei, SOX2-positive ICM cells, and CDX2-positive TE cells were counted from fluorescence images. Image acquisition and cell counting were performed by the same technician, who was blinded to the randomized treatment codes.

### 2.10. Experimental Design

Four experiments were conducted. Experiment 1 used a 2 × 2 factorial IVF design to evaluate oocyte source (SL-O or OPU-O), sperm type (conventional or X-sorted sperm), and their interaction. Experiment 2 used a one-factor-at-a-time sequential design with slaughterhouse-derived oocytes in the following order: maturation time, sperm processing, post-injection incubation medium, and activation protocol. During maturation-time screening, BO processing, OM incubation, and ETH (5) activation were held constant. During sperm-processing screening, the selected maturation time was fixed, whereas OM and ETH (5) were retained. During medium screening, the selected maturation time and sperm-processing method were used. During activation screening, the conditions selected in the preceding three steps were fixed. Within each step, all treatment levels were tested contemporaneously using oocytes from the same collection/processing batches and allocated as evenly as possible among groups. Selection followed a hierarchical decision rule: conditions with poor survival or increased abnormal, condensed-sperm-head, or nonactivation configurations were not favored; among the remaining conditions, presumptive 2PN formation and blastocyst development were prioritized; and blastocyst cell allocation was used to resolve trade-offs among activation regimens. No weighted composite score or fixed effect-size threshold was used. Experiment 3 evaluated day-7 blastocyst cell allocation and selected ETH (5) because it combined a high presumptive 2PN rate and blastocyst formation with the most favorable ICM/TE allocation among the tested regimens, without the higher frequency of abnormal or condensed-sperm-head configurations observed in some alternatives. Experiment 4 compared the complete, sequentially selected ssICSI protocol with ssIVF using OPU-derived oocytes. Following completion of the slaughterhouse-derived screening, the individual optimization steps were evaluated using OPU-derived oocytes for secondary validation (Table A3).

### 2.11. Statistical Analysis

Analyses were performed using SPSS Statistics 27.0.1 (IBM, Armonk, NY, USA). An independent experimental run was defined as one complete, separately conducted oocyte-processing and fertilization/injection session in which contemporaneous treatment groups were established from the same collection/processing batch; the run, rather than the individual oocyte, was the inferential unit. Experiment 1 was analyzed using a 2 × 2 factorial two-way analysis of variance (ANOVA), with oocyte source, sperm type, and their interaction as fixed effects. Experiments 2–4 were analyzed by one-way ANOVA using independent-run summaries as the inferential observations: run-level proportions within each sequential step for Experiment 2, treatment-specific run-level means for cell-allocation outcomes in Experiment 3, and run-level proportions for ssICSI versus ssIVF in Experiment 4. When an omnibus ANOVA was significant, Tukey’s honestly significant difference (HSD) procedure controlled multiplicity across all pairwise comparisons within that outcome and experimental step; no multiplicity adjustment was applied across biologically distinct outcomes or across separate experiments. Residual normality and homogeneity of variance were assessed using the Shapiro–Wilk and Levene tests, respectively. When assumptions were materially violated, the Kruskal–Wallis test with multiplicity-adjusted post hoc comparisons was used as the alternative analysis, not as confirmation of ANOVA. Linear regression was used only for prespecified continuous trends. Run-level proportions were retained to preserve the randomized experimental run as the unit of replication and to avoid weighting runs solely by the number of oocytes contributed, an approach also used in related bovine in vitro embryo-production studies [42,43]. Data are presented as means ± SEM of run-level proportions. Statistical significance was set at *p* < 0.05; Exact *p* values are reported in the corresponding results where available. All statistical figures were generated using Origin 2024 (OriginLab Corporation, Northampton, MA, USA).

## 3. Results

### 3.1. Sex-Sorted Sperm Exhibited a Fertilization Defect in Conventional IVF

Across both oocyte sources, X-sorted sperm produced lower 2PB extrusion (OPU-O, *p* = 0.009; SL-O, *p* < 0.001) and blastocyst rates (OPU-O, *p* < 0.001; SL-O, *p* < 0.001) than conventional sperm (Table 1; Figure 1). The 2 × 2 factorial analysis identified a strong sperm-type effect on 2PB extrusion and blastocyst formation, an oocyte-source effect on blastocyst formation, and sperm-type × oocyte-source interactions for cleavage and blastocyst formation. Pronuclear assessment showed that the presumptive normal-fertilization rate (2PN) was 2.45% with X-sorted sperm and 46.13% with conventional sperm (*p* < 0.001; Table 2). The large proportion of MII-arrested/unactivated oocytes after ssIVF identifies an early fertilization/activation deficit but does not distinguish failed zona binding, penetration, membrane fusion, activation, or sperm-head decondensation. Complete pronuclear categories are reported in Table A1.

### 3.2. Screening of Candidate Conditions Through Embryo Production Efficiency and Pronuclear Formation

In the maturation time screen, oocytes matured for 22 h produced a higher blastocyst rate (12.76%) than those matured for 18 h (4.65%, *p* < 0.001), 20 h (4.94%, *p* < 0.001), or 26 h (6.46%, *p* = 0.003) (Table 3; Figure 2). The presumptive normal fertilization (2PN) rate was also higher at 22 h (58.28%) than at 20 h (35.33%) (*p* = 0.007), with fewer abnormal fertilization and Failed Fertilization (CSH) configurations (Table 4; Figure 3). BO swim-up yielded a higher blastocyst rate than Percoll processing (10.87% vs. 4.22%, *p* = 0.023) and a higher presumptive normal fertilization (2PN) rate (58.38% vs. 46.24%, *p* = 0.018). OM produced higher 2PB extrusion (80.26% vs. 66.56%, *p* = 0.034), blastocyst formation (11.27% vs. 5.40%, *p* = 0.010), and presumptive normal fertilization (2PN) formation (62.86% vs. 42.88%, *p* = 0.009) than IVF medium.

Among activation regimens, ETH (5) produced a 2PB extrusion rate of 78.29%. ION and ION*2 produced 61.94% and 82.56%, respectively (Table 3; Figure 2). Cleavage differed among selected regimens, whereas ETH (5), ION + ETH, ION, ION*2, ION + 6D, ION*2 + 6D, and IVF did not differ in the comparisons indicated by shared superscripts. Four activation regimens produced higher blastocyst rates than IVF (5.01%): ETH (5), 10.46% (*p* = 0.008); ION + ETH, 13.96% (*p* < 0.001); ION + 6D, 15.07% (*p* < 0.001); and ION*2 + 6D, 21.14% (*p* < 0.001).

At 16 h, presumptive normal fertilization (2PN) was more frequent with ETH (5) (52.92%, *p* < 0.001), ETH (10) (39.99%, *p* < 0.001), ION + ETH (54.07%, *p* < 0.001), and ION*2 + 6D (37.55%, *p* < 0.001) than with IVF (4.05%) (Table 4; Figure 3). ION + ETH (54.07%) and ETH (5) (52.92%) had the highest presumptive 2PN values. The presumptive delayed fertilization pattern, defined as 1PN + PDSH at 16 h, was more frequent with ION + ETH (27.97%) and ETH (10) (20.71%). ION*2 + ETH showed a high frequency of condensed-sperm-head configurations (73.07%), and ION*2 + 6D showed more abnormal pronuclear-number configurations (18.61%) than several other regimens. The preliminary timing assessment showed that the frequency of condensed sperm heads had reached a low plateau by 16 h, whereas cleavage at 18 h increasingly interfered with pronuclear recognition (Table S1). Thus, 16 h provided the most discriminating fixed observation point available for separating presumptive normal fertilization from abnormal or presumptively delayed configurations. Activation regimens were advanced on the basis of 2PN formation, blastocyst development, abnormal pronuclear number or condensed-sperm-head patterns, and subsequent cell-allocation data; blastocyst rate alone was not used. Complete classifications are provided in Table A2.

### 3.3. Comparison of Blastocyst Developmental Potential Among Different Activation Protocols

Blastocyst cell-allocation results are shown in Figure 4. Total cell numbers in the IVF (189.4; *p* = 0.050 and *p* < 0.001), ETH (5) (174.8; *p* = 0.275 and *p* = 0.001), and ETH (10) (195.7; *p* = 0.031 and *p* < 0.001) groups exceeded those in the ION*2 + 6D (141.2) and ION*2 + ETH (106.4) groups, while not differing from ION + ETH (159.6). TE cell numbers in IVF (122.0; *p* = 0.003 and *p* = 0.022), ETH (5) (97.5; *p* = 0.311 and *p* = 0.730), and ETH (10) (145.2; *p* < 0.001 and *p* < 0.001) were higher than those in ION + 6D (73.3) and ION*2 + ETH (80.2); ION*2 + 6D (88.6; *p* = 0.107) was also lower than IVF. ICM cell numbers were higher in IVF (67.4) and ETH (5) (77.2) than in the other activation groups (*p* < 0.001). ETH (5) had the highest ICM/TE ratio (0.82) among the tested groups (*p* < 0.001).

### 3.4. Application of the Optimized Protocol in the Sexed-OPU-ICSI System

The combination selected in the slaughterhouse-oocyte screen (22 h maturation, BO swim-up, OM incubation, and ETH (5) activation) was evaluated using OPU-derived oocytes. Five independent ssICSI runs and seven independent ssIVF runs were included. Five ssIVF runs were contemporaneous, and oocyte collection matched to the ssICSI runs; the other two ssIVF runs were conducted on adjacent dates during the same seasonal period and were retained as independent supporting observations. Compared with ssIVF, ssICSI increased 2PB extrusion (82.31% vs. 66.00%, *p* < 0.001) and blastocyst formation (16.84% vs. 7.88%; *p* < 0.001), whereas cleavage rates were comparable (73.02% vs. 62.01%; *p* = 0.844) (Table 5). When calculated using injected oocytes as the denominator, the ssICSI blastocyst rate was 13.46%, which remained significantly different from that of ssIVF (7.88%; *p* = 0.021) (Table 5; Figure 5). OPU-based checks of individual sequential steps were performed only after the corresponding slaughterhouse-based selection decisions and are presented separately in Table A3; they did not influence selection of the final conditions.

## 4. Discussion

Using a sequential screening strategy, we identified a combination of 22 h maturation, BO swim-up, OM post-injection incubation, and ETH (5) activation that performed favorably with X-sorted sperm under the tested conditions. The selected combination increased presumptive normal fertilization (2PN) formation in the screening experiments and increased blastocyst formation in the OPU validation relative to ssIVF, while cleavage remained comparable. These findings support improved in vitro embryo production. The selected protocol was the preferred protocol identified through sequential optimization.

Conventional IVF with X-sorted sperm produced a markedly lower presumptive normal fertilization (2PN) rate and a higher proportion of unactivated (MII) oocytes than IVF with conventional sperm (Table 2 and Table A1). Previous studies have discussed the resistance of highly condensed sperm chromatin to decondensation [44,45], but the present experiment did not directly measure chromatin remodeling. Our design also did not localize spermatozoa or assess zona binding, zona penetration, membrane fusion, or post-thaw sperm quality. Therefore, the data demonstrate an early fertilization/activation deficit but do not identify failed oolemma penetration as its primary cause. The interaction between sperm type and oocyte source further indicates that the magnitude of this deficit can depend on the oocyte population, which motivated the use of abundant slaughterhouse-derived oocytes for screening and OPU-derived oocytes for subsequent validation. Because conventional and X-sorted semen came from different sets of three bulls and three coded batches per semen type, semen type could not be separated fully from bull or batch effects.

Maturation for 22 h supported higher blastocyst and presumptive normal fertilization (2PN) rates than several shorter or longer intervals (Table 3 and Table 4). Previous bovine studies reported developmental competence across approximately 22–28 h of maturation, with deterioration after more prolonged maturation [46,47,48]. Cytoplasmic glutathione can facilitate sperm-chromatin decondensation [49], and other maturation-associated factors may support male-pronucleus formation [50]. Based on the literature, we hypothesized that 22 h of maturation would provide a more favorable cytoplasmic environment.

BO swim-up produced more blastocysts and a higher presumptive normal fertilization (2PN) rate than Percoll processing (Table 3 and Table 4). BO enriches motile spermatozoa, whereas density-gradient centrifugation separates cells largely according to density [51]. Motility, chromatin integrity, and fertilizing capacity can be associated [52,53], but these attributes were not measured after processing in the present study. Recent evidence indicates that freezing and thawing can impair sperm motility, viability, plasma and acrosomal membrane integrity, mitochondrial function, and oxidative balance [54,55,56]. BO swim-up may preferentially enrich spermatozoa with superior motility and membrane integrity, thereby contributing to the improved developmental outcomes observed in this study. However, because these post-thaw sperm characteristics were not directly assessed, the cellular and molecular mechanisms underlying the beneficial effects of BO swim-up remain to be elucidated.

During post-injection activation, the use of OM as the incubation medium resulted in higher rates of second polar body extrusion, blastocyst formation, and pronuclear formation than the use of IVF medium (Table 3 and Table 4). This improvement may reflect the more favorable environment provided by OM, which may facilitate oocyte recovery following micromanipulation and support subsequent pronuclear formation and embryonic development, an important consideration in bovine ICSI [57]. Cytoskeletal reorganization, intracellular calcium homeostasis, glutathione-dependent redox regulation, and chromatin remodeling may contribute to these effects. However, because these processes were not directly assessed in this experiment, their potential roles require validation through targeted imaging and biochemical analyses.

Activation treatment influenced meiotic completion, pronuclear morphology, and blastocyst development [39,40,58]. Consistent with bovine ICSI studies by Rho et al. [39], Ock et al. [40], and Keefer et al., exogenous activation generally supported development after bovine ICSI [36,59]. Oikawa et al. reported that an ethanol-activated group had a higher calving rate than an ionomycin-followed-by-6-DMAP treatment group in a different PIEZO-assisted system [38]; this evidence is informative but cannot validate the present mechanical-injection protocol. ION*2 + 6D produced the highest blastocyst rate but also showed more condensed-sperm-head and abnormal pronuclear configurations, illustrating why blastocyst rate alone was not used to select the final regimen. ETH (5) provided a favorable balance of presumptive normal fertilization (2PN) formation, blastocyst development, and cell allocation among the tested conditions. A previous study also described developmental patterns compatible with activation without normal paternal contribution [60], and parthenogenetic development after artificial activation remains a relevant concern [61,62]. Because calcium dynamics and downstream pathways were not measured here, proposed effects on calcium signaling, glutathione, cytoskeletal recovery, or sperm-head decondensation remain literature-based hypotheses. The absence of activation-only, sham-injection-plus-activation, ICSI-without-exogenous-activation, and conventional-sperm ICSI controls prevented complete separation of sperm-dependent development from parthenogenetic activation.

ETH (5) blastocysts had the highest ICM/TE ratio among the tested groups, while total and ICM cell numbers were comparable to IVF (Figure 4). ICM and TE counts describe lineage allocation and are widely used as in vitro blastocyst-quality variables [63]. The sex-sorted-IVF ratio remained within a range reported for conventional IVF [64]. However, the higher ETH (5) ratio partly reflected fewer TE cells relative to IVF, which may indicate altered lineage allocation rather than uniformly superior development. A previous report described offspring after ethanol activation [38], but it used a different PIEZO-assisted ICSI system [65] and cannot validate the reproductive performance of the present mechanical-injection protocol. Therefore, the ICM/TE ratio was regarded as a surrogate endpoint for the in vitro experiments.

The optimized protocol significantly improved the efficiency of converting valuable gametes into viable embryos. Using OPU-derived oocytes, ssICSI increased 2PB extrusion and blastocyst formation compared with ssIVF, whereas cleavage rates did not differ significantly between the two groups (Table 5; Figure 5). Post-injection survival is an important measure of procedural efficiency because mechanical injection may cause oocyte loss before embryo culture. Although all ICSI procedures were performed by the same experienced operator, survival may still have been influenced by injection-pipette quality and inner diameter, as well as subtle variations in micromanipulation. Therefore, reporting developmental outcomes using both the number of initially injected or allocated oocytes and the number of post-procedure surviving oocytes as denominators distinguishes injection-associated losses from subsequent developmental competence and provides a more comprehensive estimate of protocol performance at the production level.

Several limitations should be emphasized. First, the four protocol components were optimized sequentially; therefore, interactions among maturation time, sperm processing, incubation medium, and activation regimen were not estimated. Second, embryo transfer, pregnancy, fetal development, and calving outcomes were not evaluated, and evidence obtained using different systems assisted by PIEZO [38] cannot establish the in vivo competence of the present protocol. Third, the study lacked comparator groups receiving activation alone, sham injection, no exogenous activation, ICSI with conventional sperm, or ICSI assisted by PIEZO [65]. Fourth, most screening experiments used oocytes derived from slaughterhouse ovaries, whereas validation using OPU involved a smaller sample. Fifth, sperm motility, viability, acrosomal integrity, mitochondrial function, and DNA and chromatin status after thawing were not independently assessed. Sixth, pronuclear classification was based on morphology at a single fixed time point. Even though 16 h minimized condensed-sperm-head configurations before extensive cleavage, rare cases of very late fertilization or unusually early pronuclear disappearance may still have been misclassified. Seventh, conventional and X-sorted semen originated from different, small sets of bulls and coded batches, preventing reliable separation of semen-type effects from bull or batch effects. Eighth, analysis of run-level proportions preserved the experimental run as the inferential unit but did not fully account for unequal binomial denominators, and the small number of runs limited reliable mixed-effects modeling. Further factorial optimization, mechanistic measurements, verification of paternal origin, and embryo transfer studies are therefore required.

## 5. Conclusions

A sequential screen identified 22 h maturation, BO swim-up, OM post-injection incubation, and ETH (5) activation as the best-performing tested combination for mechanical ICSI with X-sorted bovine sperm. The protocol increased presumptive normal fertilization (2PN) formation in the screening experiments and increased blastocyst formation in OPU-derived oocytes relative to ssIVF, while cleavage remained comparable. ETH (5) also produced a favorable ICM/TE cell allocation among the tested activation regimens. These conclusions are limited to in vitro outcomes under the tested sequential conditions. The underlying mechanisms, interactions among protocol components, paternal contribution, and in vivo competence after embryo transfer remain to be established.

## Figures and Tables

**Figure 1:**
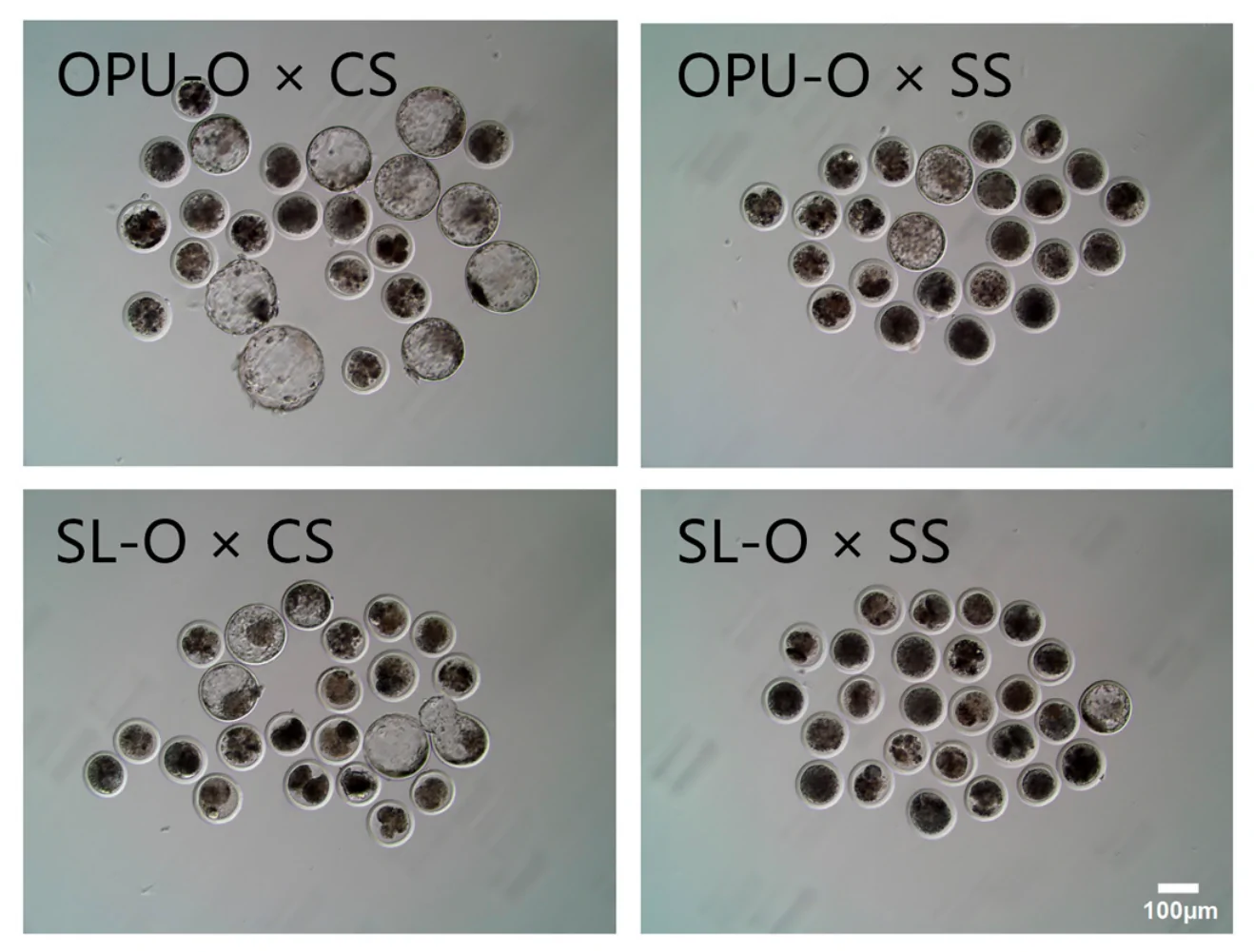
Blastocyst formation after IVF according to oocyte source and sperm type. SL-O × CS, Slaughter-house-derived oocytes with conventional sperm; SL-O × SS, Slaughterhouse-derived oocytes with sex-sorted sperm; OPU-O × CS, OPU-derived oocytes with conventional sperm; OPU-O × SS, OPU-derived oocytes with sex-sorted sperm. Scale bar = 100 µm.

**Figure 2:**
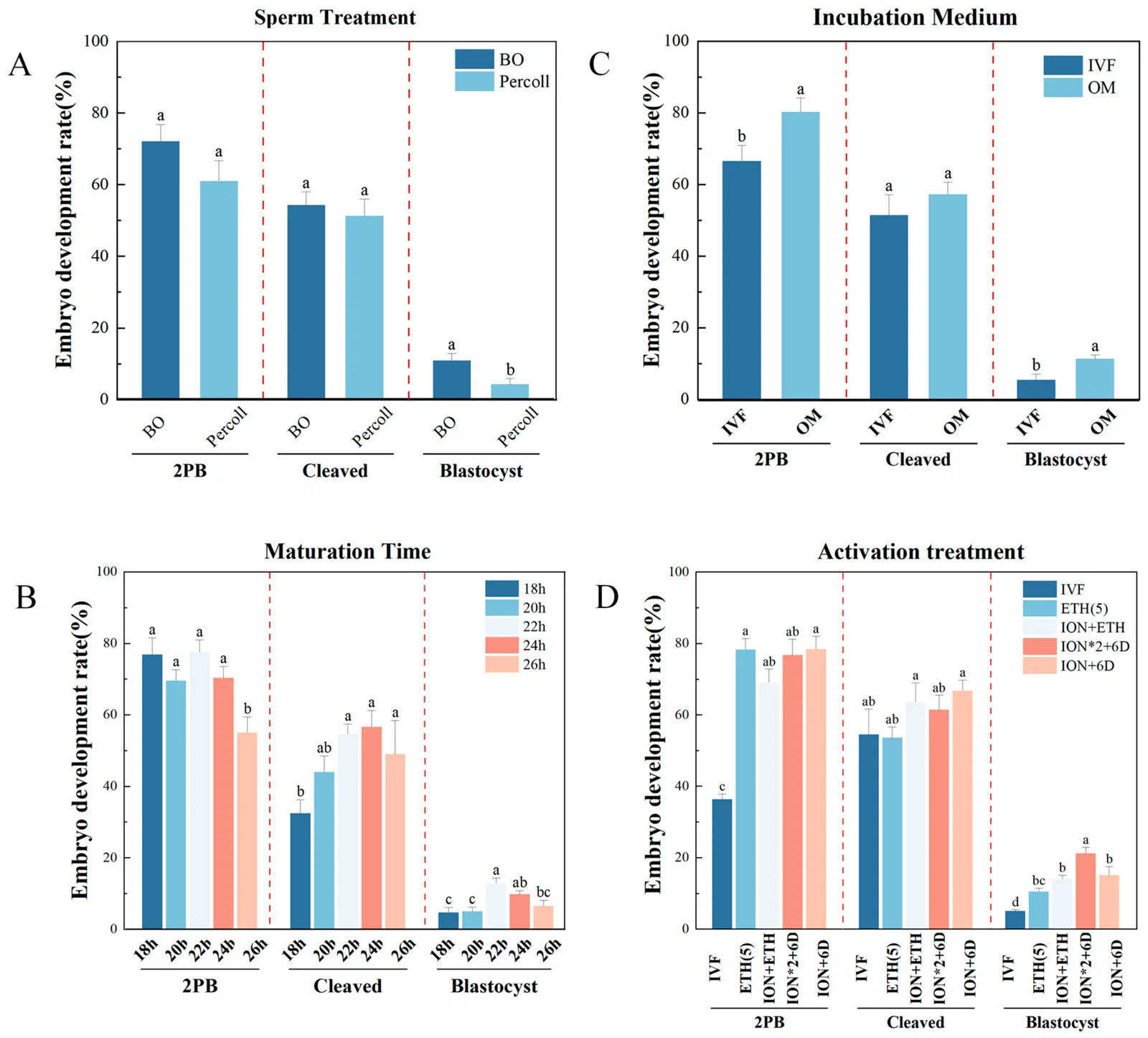
Embryo production outcomes during sequential screening of sex-sorted-sperm ICSI. (**A**) BO swim-up versus Percoll sperm processing. (**B**) OM versus IVF medium during post-injection incubation. (**C**) Oocyte maturation for 18–26 h. (**D**) Representative activation regimens that passed the initial screen (IVF, ETH (5), ION + ETH, ION + 6D, and ION*2 + 6D); complete results for all nine activation regimens and IVF are presented in Table 3. Data are means ± SEM of independent runs. Bars with different lowercase letters differ at *p* < 0.05.

**Figure 3:**
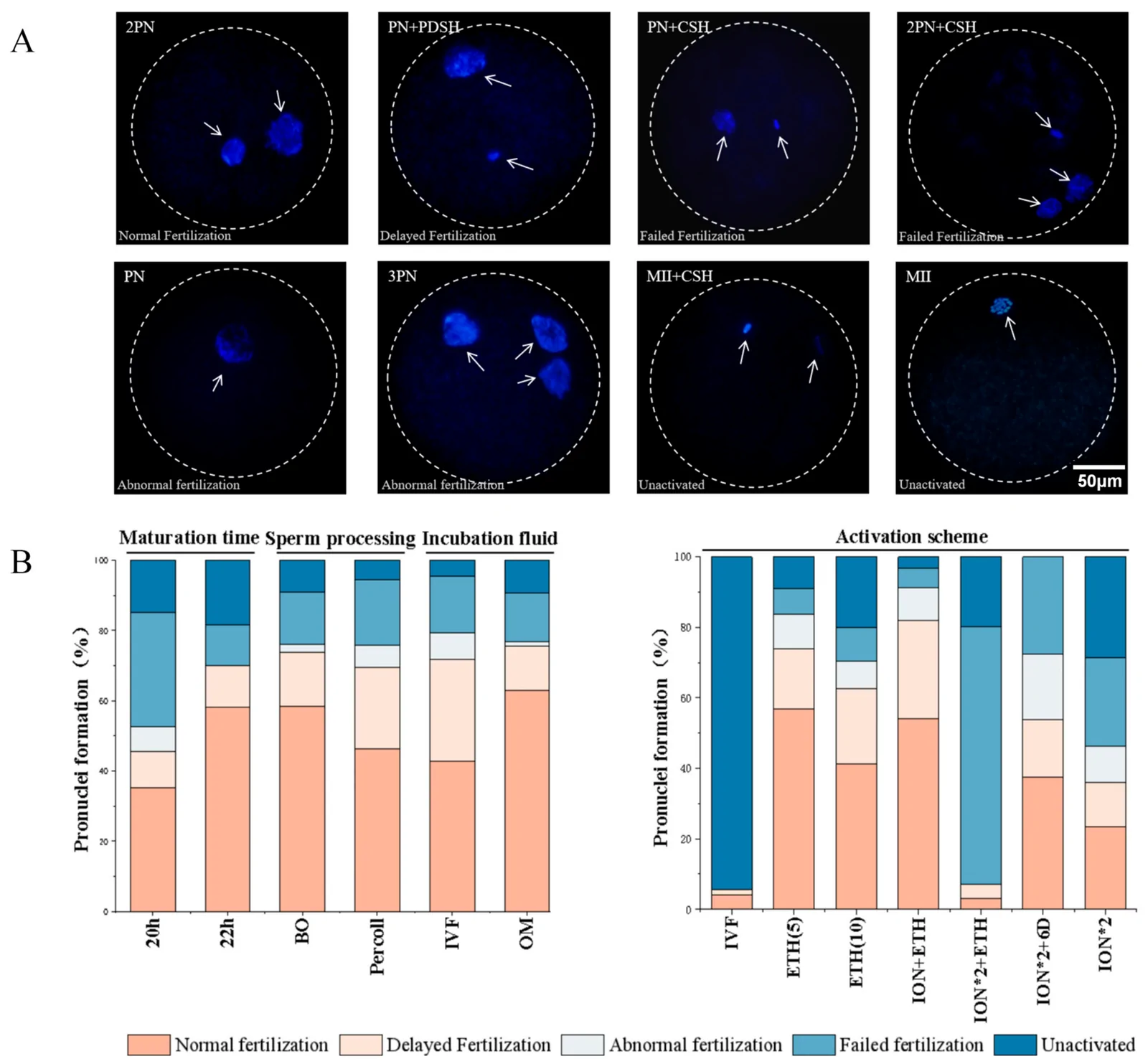
Pronuclear configurations observed during sequential screening. (**A**) Representative micrographs. 2PN, one male and one female pronucleus; 1PN + PDSH, one pronucleus with a partially decondensed sperm head; 1PN + CSH, one pronucleus with a condensed sperm head; 2PN + CSH, two pronuclei with a persistent condensed sperm head; 1PN, one pronucleus; ≥3PN, three or more pronuclei; MII and MII + CSH, MII-arrested/unactivated configurations. Scale bar = 50 µm. (**B**) Distribution of pronuclear configurations for the indicated maturation times, sperm-processing methods, incubation media, and activation regimens. At 16 h, 2PN was classified as presumptive normal fertilization; 1PN + PDSH was classified as presumptive delayed fertilization; condensed-sperm-head configurations included 1PN + CSH and 2PN + CSH; abnormal pronuclear number included 1PN and ≥3PN; and unactivated status included MII and MII + CSH. The combined proportion of 2PN and 1PN + PDSH at 16 h is reported descriptively. Circular marks indicate the boundaries of oocytes.

**Figure 4:**
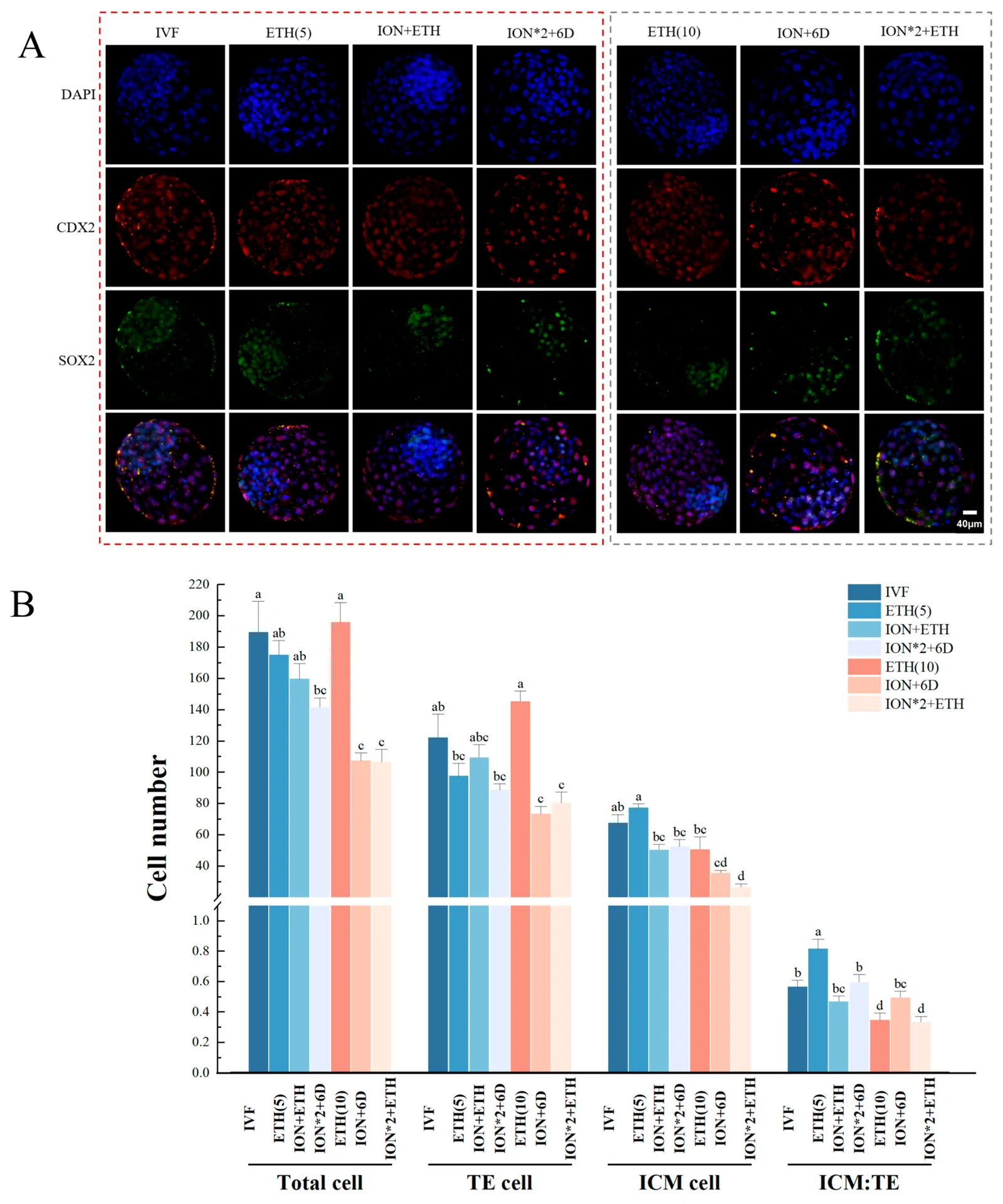
Blastocyst cell allocation after different activation regimens. (**A**) Immunofluorescence staining of total nuclei (DAPI, blue), ICM (SOX2, green), and TE (CDX2, red). Scale bar = 40 µm. Red boxes identify ETH (5), ION + ETH, ION*2 + 6D, and IVF; gray boxes identify ETH (10), ION + 6D, and ION*2 + ETH. (**B**) Total cell number, ICM cell number, TE cell number, and ICM/TE ratio. The numbers of analyzed blastocysts and independent runs were: IVF, *n* = 5 blastocysts from 5 independent runs; ETH (5), *n* = 6 blastocysts from 5 independent runs; ETH (10), *n* = 4 blastocysts from 4 independent runs; ION + ETH, *n* = 5 blastocysts from 5 independent runs; ION + 6D, *n* = 6 blastocysts from 5 independent runs; ION*2 + ETH, *n* = 5 blastocysts from 4 independent runs; and ION*2 + 6D, *n* = 5 blastocysts from 4 independent runs. Each blastocyst was an observational unit nested within an independent run; the treatment mean within each run was the inferential observation. Data are means ± SEM of run-level means. Different superscript letters indicate *p* < 0.05 by one-way ANOVA with Tukey’s HSD test, or by Kruskal–Wallis testing with multiplicity-adjusted post hoc comparisons when assumptions were not met.

**Figure 5:**
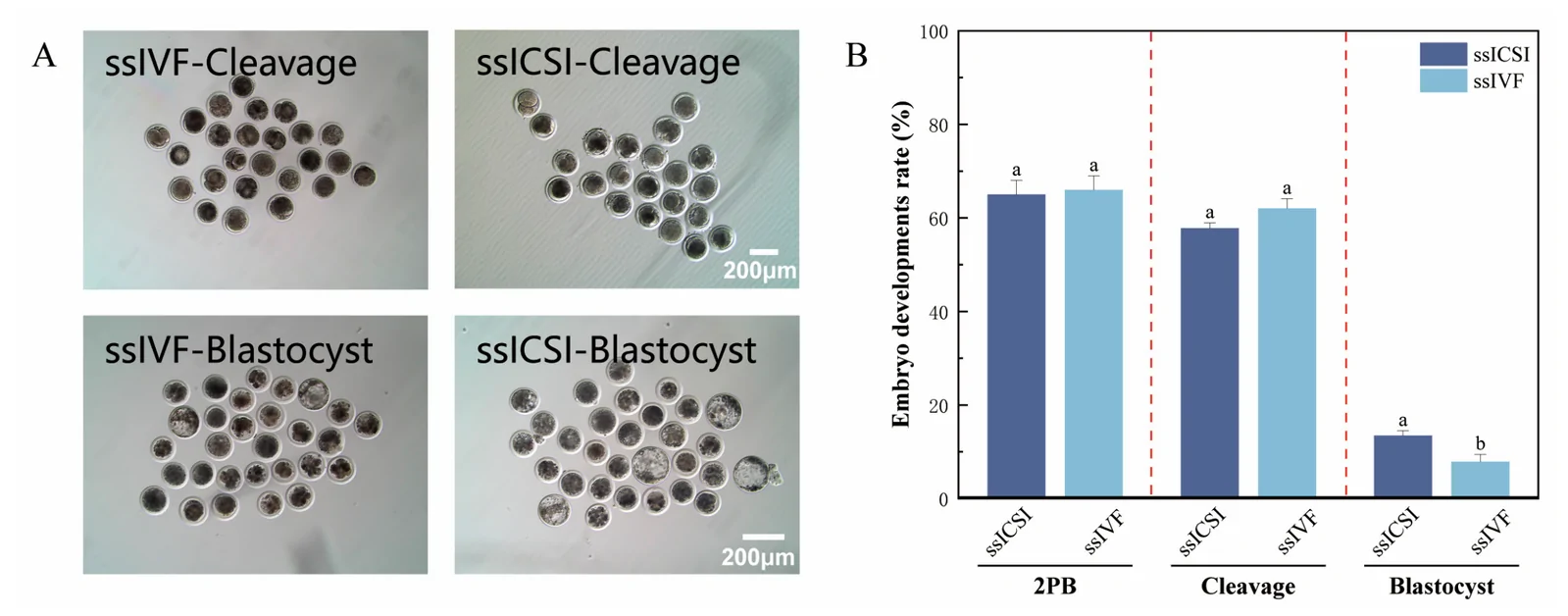
Embryo development in the OPU endpoint validation. (**A**) Representative cleavage-stage embryos (48 h; scale bar = 200 µm) and blastocysts (168 h; scale bar = 200 µm) after ssICSI or ssIVF. (**B**) Embryo-production outcomes. Data are means ± SEM of independent run-level proportions. Different letters indicate *p* < 0.05.

**Table 1:** Factorial effects of sperm type and oocyte source on IVF embryo development.

Oocyte Source	Sperm Type	No. of Oocytes	2PB Rate (%)	Cleavage Rate (%)	Blastocyst Rate (%)
OPU-O	SS	112	59.45 ± 4.03 ^b^	70.44 ± 6.50 ^a^	6.80 ± 1.71 ^b^
	CS	68	77.11 ± 3.83 ^a^	72.29 ± 3.10 ^a^	38.18 ± 5.19 ^a^
SL-O	SS	440	44.89 ± 2.40 ^b^	60.14 ± 3.80 ^b^	3.11 ± 0.59 ^b^
	CS	718	79.52 ± 4.08 ^a^	85.08 ± 2.68 ^a^	13.96 ± 1.59 ^a^
Sperm type			**	*	**
Oocyte source			NS	NS	**
Sperm type × Oocyte source			NS	*	*

Data are presented as means ± SEM of independent run-level proportions. The independent experimental run was the inferential unit. The numbers of independent runs were SL-O × conventional sperm, *n* = 5; SL-O × X-sorted sperm, *n* = 4; OPU-O × conventional sperm, *n* = 6; and OPU-O × X-sorted sperm, *n* = 8. Main effects and the interaction were evaluated by two-way ANOVA; pairwise treatment-combination comparisons used Tukey’s HSD procedure when the omnibus effect was significant. * *p* < 0.05; ** *p* < 0.001; NS, not significant. Values within the same column with the same superscript letters (e.g., a, b) are not significantly different (*p* > 0.05), while values with different superscript letters are significantly different (*p* < 0.05).

**Table 2:** Summary of pronuclear configurations after IVF with conventional or sex-sorted sperm.

Sperm Type	Stained Oocytes	Normal Fertilization	Delayed Fertilization	Abnormal Fertilization	Failed Fertilization	Unactivated
SS	82	2.45 ± 1.42 ^b^	3.56 ± 2.09 ^b^	0.00 ± 0.00 ^b^	1.14 ± 1.14 ^a^	92.85 ± 0.86 ^a^
CS	78	46.13 ± 5.76 ^a^	12.87 ± 1.59 ^a^	10.36 ± 2.23 ^a^	1.90 ± 1.19 ^a^	29.46 ± 8.87 ^b^

Data are means ± SEM from four independent runs per sperm type. Different superscript letters within a column indicate *p* < 0.05 by one-way ANOVA with Tukey’s HSD test. Presumptive normal fertilization was defined morphologically as 2PN; 1PN + PDSH at 16 h was classified as presumptive delayed fertilization; condensed-sperm-head configurations included 1PN + CSH and 2PN + CSH; abnormal pronuclear number included 1PN and ≥3PN; and unactivated status included MII and MII + CSH. The combined proportion of 2PN and 1PN + PDSH at 16 h is reported descriptively.

**Table 3:** Embryo production efficiency at different optimization stages.

Optimization Parameter		No. of Injected	No. of Surviving	2PB Rate (%)	Cleavage Rate (%)	Blastocyst Rate (%)
Maturation time	18 h	216	165	76.85 ± 4.68 ^a^	32.41 ± 3.80 ^b^	4.65 ± 1.36 ^c^
	20 h	263	204	69.57 ± 3.02 ^a^	43.97 ± 4.48 ^ab^	4.94 ± 1.18 ^c^
	22 h	201	167	77.61 ± 3.26 ^a^	54.60 ± 2.77 ^a^	12.76 ± 1.45 ^a^
	24 h	204	161	70.30 ± 3.20 ^a^	56.60 ± 4.58 ^a^	9.74 ± 0.94 ^ab^
	26 h	202	161	54.99 ± 4.33 ^b^	48.93 ± 9.45 ^a^	6.46 ± 1.57 ^bc^
Sperm treatment	BO	165	127	72.04 ± 4.70 ^a^	54.27 ± 3.61 ^a^	10.87 ± 1.93 ^a^
	Percoll	164	120	60.96 ± 5.67 ^a^	51.29 ± 4.61 ^a^	4.22 ± 1.56 ^b^
Incubation medium	OM	217	143	80.26 ± 3.76 ^a^	57.24 ± 3.30 ^a^	11.27 ± 1.11 ^a^
	IVF	183	136	66.56 ± 4.30 ^b^	51.41 ± 5.64 ^a^	5.40 ± 1.58 ^b^
Activation treatment	ETH(5)	279	203	78.29 ± 3.07 ^a^	53.56 ± 2.99 ^ab^	10.46 ± 0.99 ^bc^
	ETH(10)	144	120	74.23 ± 2.87 ^ab^	42.84 ± 4.96 ^b^	5.83 ± 1.13 ^cd^
	ION + ETH	227	169	69.07 ± 3.68 ^ab^	63.51 ± 5.42 ^a^	13.96 ± 1.06 ^b^
	ION*2 + ETH	113	86	61.68 ± 5.64 ^b^	42.87 ± 3.50 ^b^	4.15 ± 1.85 ^d^
	ION + 6D	100	80	78.33 ± 3.64 ^a^	66.76 ± 2.92 ^a^	15.07 ± 2.39 ^b^
	ION*2 + 6D	206	161	76.70 ± 4.40 ^ab^	61.46 ± 3.98 ^ab^	21.14 ± 1.67 ^a^
	ION	94	82	61.94 ± 7.99 ^b^	46.93 ± 11.43 ^ab^	4.67 ± 2.12 ^d^
	ION*2	116	90	82.56 ± 5.60 ^a^	49.89 ± 4.31 ^ab^	6.61 ± 2.17 ^cd^
	EA	127	111	68.99 ± 7.79 ^ab^	49.24 ± 10.98 ^ab^	2.50 ± 1.15 ^d^
	IVF	-	1201	36.38 ± 1.42 ^c^	54.50 ± 7.05 ^ab^	5.01 ± 0.40 ^d^

Data are means ± SEM of independent run-level proportions. ICSI survival was calculated relative to initially injected oocytes; subsequent developmental rates were calculated relative to post-injection survivors to describe competence after technically successful injection. The independent run was the inferential unit. The numbers of independent runs were: maturation time, 18 h (*n* = 9), 20 h (*n* = 12), 22 h (*n* = 9), 24 h (*n* = 7), and 26 h (*n* = 7); sperm processing, BO (*n* = 6) and Percoll (*n* = 6); incubation medium, IVF (*n* = 7) and OM (*n* = 7); and activation treatment, IVF (*n* = 7), ETH (5) (*n* = 10), ETH (10) (*n* = 7), ION + ETH (*n* = 10), ION*2 + ETH (*n* = 5), ION + 6D (*n* = 5), ION*2 + 6D (*n* = 10), ION (*n* = 5), ION*2 (*n* = 7), and EA (*n* = 6). Different superscript letters within an outcome and optimization step indicate *p* < 0.05 using one-way ANOVA with Tukey’s HSD test, or Kruskal–Wallis testing with multiplicity-adjusted post hoc comparisons when model assumptions were not met.

**Table 4:** Summary of pronuclear configurations at each sequential screening step.

Optimization Parameter		Stained Oocytes	Normal Fertilization	Delayed Fertilization	Abnormal Fertilization	Failed Fertilization	Unactivated
Maturation time	20 h	45	35.33 ± 5.19 ^b^	10.26 ± 4.24 ^a^	7.16 ± 2.92 ^a^	32.34 ± 2.60 ^a^	14.92 ± 2.05 ^a^
	22 h	44	58.28 ± 4.63 ^a^	11.69 ± 2.74 ^a^	0.00 ± 0.00 ^b^	11.69 ± 4.00 ^b^	18.35 ± 4.24 ^a^
Sperm treatment	BO	59	58.38 ± 2.98 ^a^	15.53 ± 1.49 ^b^	2.08 ± 2.08 ^a^	14.83 ± 2.18 ^a^	9.19 ± 3.51 ^a^
	Percoll	65	46.24 ± 2.34 ^b^	23.37 ± 2.20 ^a^	6.30 ± 2.34 ^a^	18.62 ± 2.93 ^a^	5.49 ± 2.15 ^a^
Incubation medium	OM	54	62.86 ± 4.99 ^a^	12.68 ± 2.21 ^b^	1.32 ± 1.32 ^a^	13.70 ± 2.74 ^a^	9.45 ± 4.59 ^a^
	IVF	63	42.88 ± 1.45 ^b^	28.96 ± 0.85 ^a^	7.61 ± 5.36 ^a^	16.14 ± 4.76 ^a^	4.42 ± 2.60 ^a^
Activation treatment	ETH(5)	45	52.92 ± 1.77 ^a^	15.85 ± 2.62 ^b^	9.06 ± 0.67 ^bc^	6.79 ± 2.36 ^d^	8.35 ± 3.00 ^bcd^
	ETH(10)	40	39.99 ± 1.87 ^b^	20.71 ± 4.25 ^ab^	7.48 ± 2.65 ^bc^	9.22 ± 3.47 ^cd^	19.48 ± 2.71 ^b^
	ION + ETH	50	54.07 ± 2.11 ^a^	27.97 ± 2.26 ^a^	9.21 ± 3.80 ^bc^	5.63 ± 3.29 ^d^	3.14 ± 1.82 ^cd^
	ION*2 + ETH	47	3.03 ± 3.03 ^d^	4.01 ± 2.23 ^cd^	0.00 ± 0.00 ^c^	73.07 ± 2.13 ^a^	19.88 ± 3.21 ^bc^
	ION*2 + 6D	49	37.55 ± 4.09 ^b^	16.28 ± 2.27 ^b^	18.61 ± 3.79 ^a^	27.56 ± 5.87 ^b^	0.00 ± 0.00 ^d^
	ION*2	47	23.44 ± 1.88 ^c^	12.44 ± 4.00 ^bc^	10.50 ± 3.20 ^ab^	25.11 ± 9.04 ^bc^	28.50 ± 11.06 ^b^
	IVF	104	4.05 ± 1.40 ^d^	1.67 ± 1.08^d^	0.00 ± 0.00 ^c^	0.00 ± 0.00 ^d^	94.29 ± 2.08 ^a^

Data are means ± SEM of run-level proportions; the number of stained oocytes is reported for each treatment in the table. The independent run was the inferential unit. The numbers of independent runs were: maturation time, 20 h (*n* = 4) and 22 h (*n* = 4); sperm processing, BO (*n* = 4) and Percoll (*n* = 4); incubation medium, IVF (*n* = 4) and OM (*n* = 4); and activation treatment, IVF (*n* = 4), ETH (5) (*n* = 4), ETH (10) (*n* = 4), ION + ETH (*n* = 4), ION*2 + ETH (*n* = 3), ION*2 + 6D (*n* = 5), and ION*2 (*n* = 5). Different superscript letters within a column and optimization step indicate *p* < 0.05 using one-way ANOVA with Tukey’s HSD test, or Kruskal–Wallis testing with multiplicity-adjusted post hoc comparisons when assumptions were not met.

**Table 5:** Endpoint comparison of ssICSI and ssIVF using OPU-derived oocytes.

Optimization Parameter		No. of Injected	No. of Surviving	2PB/Injected (%)	2PB/Surviving (%)	Cleavage/ Injected (%)	Cleavage/ Surviving (%)	Blastocyst/ Injected (%)	Blastocyst/ Surviving (%)
ssICSI	22h	97	77	65.03 ± 3.01 ^a^	82.31 ± 6.42 ^a^	57.78 ± 1.15 ^a^	73.02 ± 4.30 ^a^	13.46 ± 1.03 ^a^	16.84 ± 1.08 ^a^
	BO								
	OM								
	ETH (5)								
ssIVF		-	101	66.00 ± 2.97 ^a^	66.00 ± 2.97 ^b^	62.01 ± 2.11 ^a^	62.01 ± 2.11 ^a^	7.88 ± 1.54 ^b^	7.88 ± 1.54 ^b^

Data are means ± SEM of independent run-level proportions: ssICSI, *n* = 5; ssIVF, *n* = 7. For ssICSI, survival was calculated relative to the number of injected oocytes, and developmental outcomes were calculated using both injected and post-injection surviving oocytes as denominators. For ssIVF, the oocyte count refers to the number retained for culture and therefore represents the initially allocated denominator. The independent run was the inferential unit, and the two groups were compared by one-way ANOVA of run-level proportions or by Kruskal–Wallis testing when assumptions were not met. Different superscript letters within a column indicate *p* < 0.05.

## Data Availability

The original contributions presented in the study are included in the article/Supplementary Materials; further inquiries can be directed to the corresponding authors.

## Supplementary Materials

The following Supporting Information can be downloaded at: https://www.mdpi.com/article/10.3390/ani16192983/s1, Table S1: The dynamics of pronuclear formation at different time points following ICSI.

## Abbreviations

The following abbreviations are used in this manuscript:

ICSI	Intracytoplasmic Sperm Injection
OPU	Ovum Pick-up
OM	Oocyte Maturation
IVF	In Vitro Fertilization
IVC	In Vitro Culture
OPU-O	OPU-derived Oocytes
SL-O	Slaughterhouse-derived Oocytes
SS	Sex-sorted Sperm
CS	Conventional Sperm
COCs	Cumulus-oocyte Complexes
ETH	Ethanol
ION	Ionomycin
6D	6-Dimethylaminopurine
EA	Electrical Activation
PB	Polar Body
PN	Pronuclei
PDSH	Partially Decondensed Sperm Head
CSH	Condensed Sperm Head
ssICSI	Sex-sorted Sperm Intracytoplasmic Sperm Injection
ssIVF	Sex-sorted Sperm In Vitro Fertilization

## Appendix A

**Table A1:** Complete pronuclear classification after IVF with conventional or sex-sorted sperm.

Sperm Type	Stained Oocytes	2PN	1PN + PDSH	1PN + CSH	1PN	nPN (*n* ≥ 3)	2PN + CSH	MII (+CSH)
SS	82	2.45 ± 1.42 ^b^	3.56 ± 2.09 ^b^	1.14 ± 1.14 ^a^	0.00 ± 0.00	0.00 ± 0.00 ^b^	0.00 ± 0.00	92.85 ± 0.86 ^a^
CS	78	46.13 ± 5.76 ^a^	12.87 ± 1.59 ^a^	1.19 ± 1.19 ^a^	0.00 ± 0.00	10.36 ± 2.23 ^a^	0.00 ± 0.00	29.46 ± 8.87 ^b^

Data are presented as means ± SEM of independent run-level proportions. Different letters within the same column indicate significant differences (*p* < 0.05), whereas identical letters indicate no significant difference (*p* > 0.05). The independent experimental run was the inferential unit. The numbers of independent runs were conventional sperm, *n* = 4, and X-sorted sperm, *n* = 4. Significance was evaluated by one-way ANOVA with Tukey’s HSD post hoc test or by Kruskal–Wallis testing with multiplicity-adjusted post hoc comparisons when ANOVA assumptions were materially violated.

**Table A2:** Complete classification of pronuclear types at different optimization steps.

Optimization Parameter		Stained Oocytes	2PN	1PN + PDSH	1PN + CSH	1PN	nPN (*n* ≥ 3)	2PN + CSH	MII (+CSH)
Maturation time	20h	45	35.33 ± 5.19 ^b^	10.26 ± 4.24 ^a^	29.00 ± 0.90 ^a^	3.57 ± 3.57 ^a^	3.59 ± 2.08 ^a^	3.33 ± 3.33 ^a^	14.92 ± 2.05 ^a^
	22h	44	58.28 ± 4.63 ^a^	11.69 ± 2.74 ^a^	11.69 ± 4.00 ^b^	0.00 ± 0.00 ^a^	0.00 ± 0.00 ^a^	0.00 ± 0.00 ^a^	18.35 ± 4.24 ^a^
Sperm treatment	BO	59	58.38 ± 2.98 ^a^	15.53 ± 1.49 ^b^	9.80 ± 2.36 ^a^	2.08 ± 2.08 ^a^	0.00 ± 0.00 ^a^	5.02 ± 2.98 ^a^	9.19 ± 3.51 ^a^
	Percoll	65	46.24 ± 2.34 ^b^	23.37 ± 2.20 ^a^	12.10 ± 1.90 ^a^	4.02 ± 2.53 ^a^	2.27 ± 2.27 ^a^	6.52 ± 2.43 ^a^	5.49 ± 2.15 ^a^
Incubation medium	OM	54	62.86 ± 4.99 ^a^	12.68 ± 2.21 ^a^	11.07 ± 1.23 ^a^	1.32 ± 1.32 ^a^	0.00 ± 0.00 ^a^	2.63 ± 2.63 ^a^	9.45 ± 4.59 ^a^
	IVF	63	42.88 ± 1.45 ^b^	28.96 ± 0.85 ^a^	9.47 ± 0.72 ^a^	3.06 ± 1.88 ^a^	4.55 ± 4.55 ^a^	6.67 ± 4.08 ^a^	4.42 ± 2.60 ^a^
Activation treatment	ETH(5)	45	52.92 ± 1.77 ^a^	15.85 ± 2.62 ^b^	1.79 ± 1.79^c^	6.79 ± 2.36 ^ab^	2.27 ± 2.27 ^bc^	5.00 ± 2.89 ^bc^	8.35 ± 3.00 ^bcd^
	ETH(10)	40	39.99 ± 1.87 ^b^	20.71 ± 4.25 ^ab^	4.86 ± 4.86^c^	4.36 ± 2.52 ^ab^	3.13 ± 3.13 ^bc^	4.36 ± 2.52 ^bc^	19.48 ± 2.71 ^b^
	ION + ETH	50	54.07 ± 2.11 ^a^	27.97 ± 2.26 ^a^	5.63 ± 3.29 ^c^	1.47 ± 1.47 ^b^	7.73 ± 2.88 ^ab^	0.00 ± 0.00^c^	3.14 ± 1.82 ^cd^
	ION*2 + ETH	47	3.03 ± 3.03 ^d^	4.01 ± 2.23 ^cd^	36.53 ± 1.07 ^a^	0.00 ± 0.00 ^b^	0.00 ± 0.00 ^c^	36.53 ± 1.07 ^a^	19.88 ± 3.21 ^bc^
	ION*2 + 6D	49	37.55 ± 4.09 ^b^	16.28 ± 2.27 ^b^	15.56 ± 4.96 ^bc^	8.22 ± 3.78 ^ab^	10.39 ± 3.24 ^a^	12.00 ± 5.61 ^b^	0.00 ± 0.00 ^d^
	ION*2	47	23.44 ± 1.88^c^	12.44 ± 4.00 ^bc^	25.11 ± 9.04 ^ab^	10.50 ± 3.20 ^a^	0.00 ± 0.00 ^c^	0.00 ± 0.00 ^c^	28.50 ± 11.06 ^b^
	IVF	104	4.05 ± 1.40 ^d^	1.67 ± 1.08 ^d^	0.00 ± 0.00 ^c^	0.00 ± 0.00 ^b^	0.00 ± 0.00 ^c^	0.00 ± 0.00 ^c^	94.29 ± 2.08 ^a^

Data are presented as means ± SEM of independent run-level proportions. Different letters within the same column and optimization step indicate significant differences (*p* < 0.05), whereas identical letters indicate no significant difference (*p* > 0.05). The independent experimental run was the inferential unit. The numbers of independent runs were: maturation time, 20 h (*n* = 4) and 22 h (*n* = 4); sperm processing, BO (*n* = 4) and Percoll (*n* = 4); incubation medium, IVF (*n* = 4) and OM (*n* = 4); and activation treatment, IVF (*n* = 4), ETH (5) (*n* = 4), ETH (10) (*n* = 4), ION + ETH (*n* = 4), ION*2 + ETH (*n* = 3), ION*2 + 6D (*n* = 5), and ION*2 (*n* = 5). Significance was evaluated separately within each optimization step by one-way ANOVA with Tukey’s HSD post hoc test or by Kruskal–Wallis testing with multiplicity-adjusted post hoc comparisons when ANOVA assumptions were materially violated.

## Appendix B

**Table A3:** Embryo production efficiency at different optimization stages for OPU-derived oocytes.

Optimization Parameter		*n* of Oocytes Injected	*n* of Surviving Oocytes	2PB Rate (%)	Cleavage Rate (%)	Blastocyst Rate (%)
Maturation time	18 h	68	53	58.20 ± 3.71 ^b^	33.60 ± 3.49 ^a^	5.43 ± 2.29 ^b^
	20 h	86	69	69.92 ± 6.55 ^ab^	48.90 ± 7.40 ^a^	9.19 ± 2.68 ^ab^
	22 h	118	103	84.91 ± 4.25 ^a^	61.99 ± 9.19 ^a^	14.94 ± 1.64 ^a^
	24 h	144	134	80.08 ± 5.11 ^a^	54.02 ± 8.60 ^a^	10.70 ± 1.00 ^ab^
	26 h	90	61	59.03 ± 3.81 ^b^	44.81 ± 8.60 ^a^	6.74 ± 2.99 ^b^
Sperm treatment	BO	80	63	87.04 ± 7.02 ^a^	57.43 ± 2.10 ^a^	16.94 ± 2.02 ^a^
	Percoll	101	86	85.35 ± 5.05 ^a^	73.96 ± 9.17 ^a^	8.20 ± 2.42 ^b^
Incubation medium	OM	89	70	84.49 ± 3.15 ^a^	63.22 ± 8.98 ^a^	14.37 ± 2.97 ^a^
	IVF	78	59	82.71 ± 5.94 ^a^	65.71 ± 13.21 ^a^	6.80 ± 1.70 ^b^
Activation treatment	ETH(5)	97	77	82.31 ± 6.42 ^a^	73.02 ± 4.30 ^a^	16.84 ± 1.08 ^a^
	IVF	-	101	66.00 ± 2.97 ^b^	62.01 ± 2.11 ^a^	7.88 ± 1.54 ^b^

Data are presented as means ± SEM of independent run-level proportions. These OPU-derived observations were obtained after completion of the corresponding slaughterhouse-based screening decisions and did not influence selection of the final protocol. Different letters within the same column and validation stage indicate significant differences (*p* < 0.05), whereas identical letters indicate no significant difference (*p* > 0.05). The independent experimental run was the inferential unit. The numbers of independent runs were: maturation time, 18 h (*n* = 5), 20 h (*n* = 5), 22 h (*n* = 8), 24 h (*n* = 7), and 26 h (*n* = 5); sperm processing, BO (*n* = 6) and Percoll (*n* = 6); incubation medium, IVF (*n* = 5) and OM (*n* = 5); and activation treatment, IVF (*n* = 7) and ETH (5) (*n* = 5). Significance was evaluated separately within each validation stage by one-way ANOVA with Tukey’s HSD post hoc test or by Kruskal–Wallis testing with multiplicity-adjusted post hoc comparisons when ANOVA assumptions were materially violated.
