# Supplementary material for: Optimization of a Bovine Intracytoplasmic Sperm Injection System Using Sex-Sorted Sperm: Screening with Slaughterhouse-Derived Oocytes and Validation with Ovum Pick-Up-Derived Oocytes
Source: Animals (Basel). 2026 Sep 22;16(19):2983. doi: 10.3390/ani16192983 (PMC13663371; doi:10.3390/ani16192983)
Supplement: Supplementary file 1 [file animals-16-02983-s001.zip › animals-4528079-supplementary.pdf]

Supplementary Materials

Supplementary Table S1. The dynamics of pronuclear formation at different time points following ICSI.

Time	No. of oocytes	CSH+PN	PDSH+PN	2PN	1PN/≥3PN	MⅡ	Degenerated	Cleaved	Remarks
8h	30	40.00%	10.00%	16.67%	16.67%	13.33%	3.33%	0.00%	Too early at this time point: part of the sperm has not yet completed deagglutination.
10h	30	40.00%	10.00%	20.00%	10.00%	20.00%	0.00%	0.00%	CSH remains at a high level.
12h	30	33.33%	13.33%	13.33%	20.00%	10.00%	10.00%	0.00%	The probability of observing CSH decreases.
14h	32	21.88%	28.13%	9.38%	21.88%	9.38%	6.25%	3.13%	The probability of observing CSH decreases further.
16h	30	6.67%	23.33%	26.67%	10.00%	16.67%	3.33%	13.33%	The optimal observation time point: the probability of observing CSH has dropped to the bottom, and the cleavage rate is still low.
18h	39	7.69%	15.38%	23.08%	12.82%	10.26%	10.26%	20.51%	Late stage: CSH no longer decreased. A large number of cleaved embryos interfered with PN recognition.

Note: CSH = condensed sperm head (spermatozoon has not yet initiated decondensation); PDSH = partially decondensed sperm head (decondensation is in progress and pronucleus has not yet formed); 2PN = two pronuclei (two distinguishable pronuclei have been fully formed); 1PN/≥3PN = only one pronucleus present or three or more pronuclei observed; MⅡ = condensed oocyte-originated chromatin detected, oocyte remains unactivated; Degenerated = no distinguishable structures observable inside the ooplasm; Cleaved embryo = embryo that has completed the first cleavage division.

Data were obtained from 3 independent biological replicates, and each replicate used oocytes collected from the same slaughterhouse-derived batch. Pooled data are presented as mean percentages. Pronuclear formation status observed at 6 different time points was statistically evaluated in this pre-experiment.

The proportion of CSH showed a declining trend from 14 h to 16 h (dropped from 21.88% to 6.67%), and remained stable at 18 h (7.69%) without further reduction. In addition, the cleavage rate (20.51%) at 18 h would interfere with the statistical identification of pronuclei. Therefore, 16 h was selected as the sampling time point for pronuclear evaluation.
